# Higher COVID-19 mortality in low-income communities in the City of Cape Town – a descriptive ecological study

**DOI:** 10.12688/gatesopenres.13288.1

**Published:** 2021-06-04

**Authors:** Hannah Hussey, Nesbert Zinyakatira, Erna Morden, Muzzammil Ismail, Masudah Paleker, Jamy-Lee Bam, Leslie London, Andrew Boulle, Mary-Ann Davies

**Affiliations:** 1 Health Intelligence, Western Cape Government: Health, Cape Town, South Africa; 2 School of Public Health and Family Medicine, University of Cape Town, Cape Town, South Africa; 3 Division of Health Systems and Public Health, Stellenbosch University, Cape Town, South Africa

**Keywords:** COVID-19, mortality, low-income

## Abstract

Background

Cape Town, a South African city with high levels of economic inequality, has gone through two COVID-19 waves. There is evidence globally that low-income communities experience higher levels of morbidity and mortality during the pandemic.

Methods

Age-standardized COVID-19 mortality in the eight sub-districts of Cape Town was compared by economic indicators taken from the most recent Census (unemployment rate, monthly income).

Results

The overall Standardized Death Rate (SDR) for COVID-19 in Cape Town was 1 640 per million, but there was wide variation across the different sub-districts. A linear relationship was seen between sub-districts with high poverty and high COVID-19 SDRs.

Conclusions

Low-income communities in Cape Town experienced higher levels of COVID-19 mortality. As we continue to contend with COVID-19, these communities need to be prioritized for access to quality health care.

## Introduction

South Africa is the most unequal country in the world, with a Gini Coefficient of 0.63 ^1, 2^, and within its cities the same inequities prevail. Post-apartheid Cape Town is a highly divided and unequal city, with socio-economic characteristics still differing along geographic lines ^3^. In 2016, the city’s population was 4 232 276 and the Gini coefficient was 0.61 ^4^.

Since SARS-CoV-2 reached Cape Town in March 2020, the city has experienced two waves of COVID-19. The first wave peaked in June-July 2020, and the second more severe wave peaked in December 2020 – January 2021, and was driven largely by the new 501.YV2 (B1.351) variant ^5, 6^.

Data from the United States (USA) has shown that low socio-economic status, and race as its proxy, were significantly associated with COVID-19 incidence and mortality ^7^. Similar findings have been reported in the United Kingdom (UK) ^8– 10^. Data from Brazil and Mexico further support the idea that the poorest population groups have lower survival from COVID-19 ^9, 10^.

**Figure 1.**
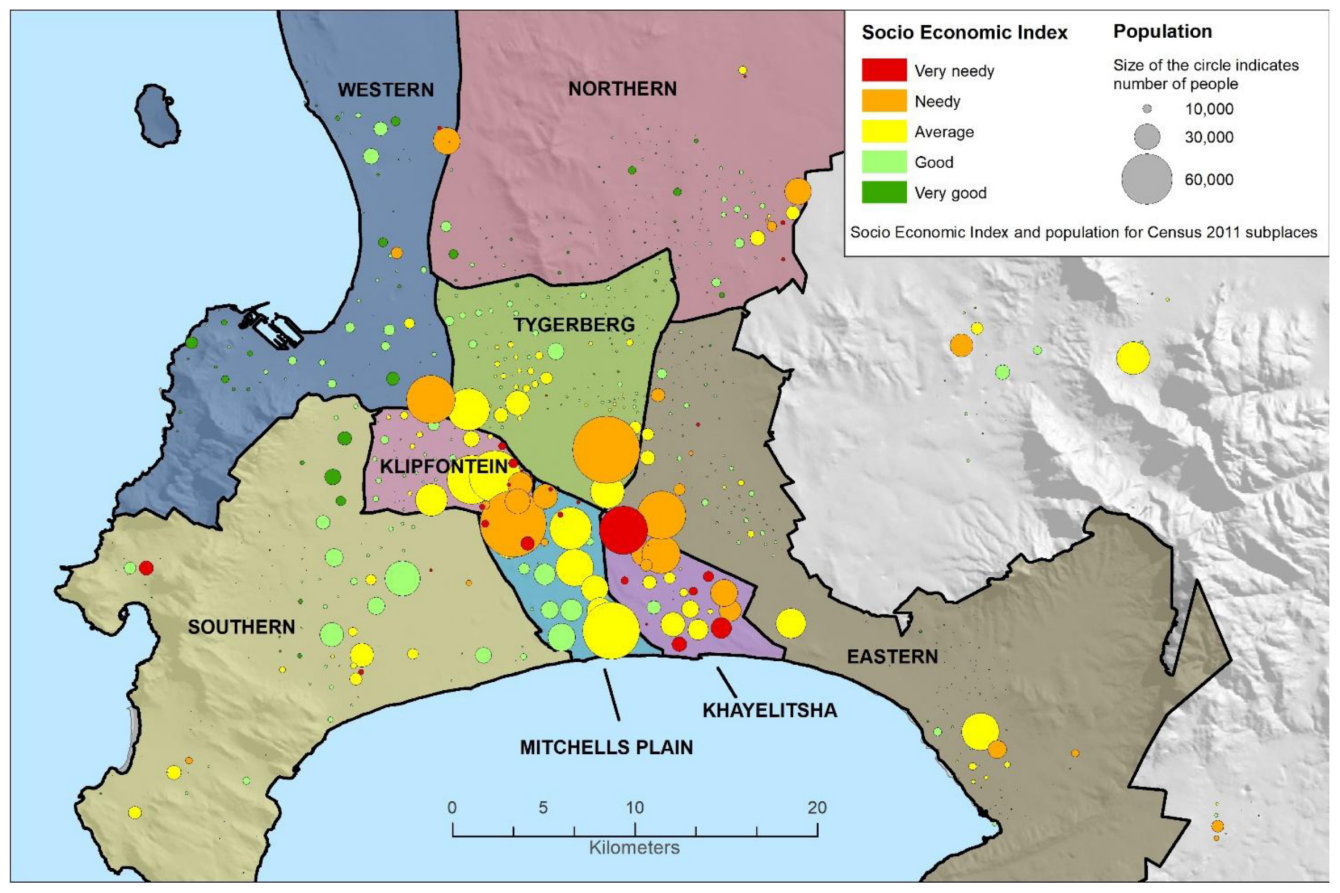
Map of Cape Town Metro, with socio-economic index and population size taken from the 2011 Census ^3^. Graphic courtesy of the Western Cape Department of Health.

Cape Town Metro has eight geographic sub-districts. Due to the city’s apartheid history, inequity tends to follow these geographic boundaries and the majority of the population resides in the historically Black African and Coloured (mixed ancestry) areas towards the southeast of the city (see Figure 1 map) ^11^. There is, however, still some heterogeneity within sub-districts. These low-income communities also tend to be overcrowded and denser, with less access to services like running water and electricity ^2^. The populations in these areas are also younger, with for instance 1.6% of the population in Khayelitsha being over 65 years of age, compared to 7.3% in Northern ^3^.

Since older age is the strongest risk factor for COVID-19 mortality ^12^, crude mortality rates may mask inequities in COVID-19 outcomes as poorer populations tend to be younger. We therefore aimed to compare the age- and sex-standardized mortality in different geographical areas of Cape Town known to have different income levels.

## Methods

This descriptive ecological study was done using aggregate data routinely reported as part of the Western Cape Department of Health’s COVID-19 Surveillance Response, from 1 March 2020 to 28 February 2021. The main outcome of interest was the COVID-19 standardized death rate (SDR) and the main exposures were the economic indicators, for each sub-district. COVID-19 deaths include all deaths ascertained by the services, or through linkage to the national population register in patients with civil identifiers available, in individuals with laboratory confirmed COVID-19 (either PCR or antigen positive). This is limited to deaths within 28-days of a COVID-19 diagnosis or within 14-days of being discharged following admission due to COVID-19, and excludes deaths manually flagged as incidental by case managers or identified as non-natural deaths on the population register.

COVID-19 tests are performed in either the public National Health Laboratory Service or in various private laboratories. The Standardized Death Rate (SDR) is calculated by adjusting for the age and sex breakdown of each sub-district in the province, using the Western Cape Province as the standard population, using Microsoft Excel.

Data on economic indicators was taken from the latest Census in 2011, which is publicly available online ^3^. Unemployment was defined as “persons who did not work, but who looked for work and were available to work in the reference period”, and the labor force was defined as persons aged 15–64 years ^3^. The 2011 Census classified households with monthly incomes below ZAR3200, which when converted at the average 2011 exchange rate is around US$ 440 ^13^.

The co-morbidities amongst public sector patients are primarily reported from inferred health episodes at the Provincial Health Data Centre, where routine health data, such as laboratory tests and medication dispensed, is used to infer health conditions ^14^. The co-morbidities of private patients are reliant on manual capture, and there is the risk of under-ascertainment in this latter group.

No ethics approval was sought for this study, as it relies upon aggregate data from routine reports.

## Results

**Figure 2.**
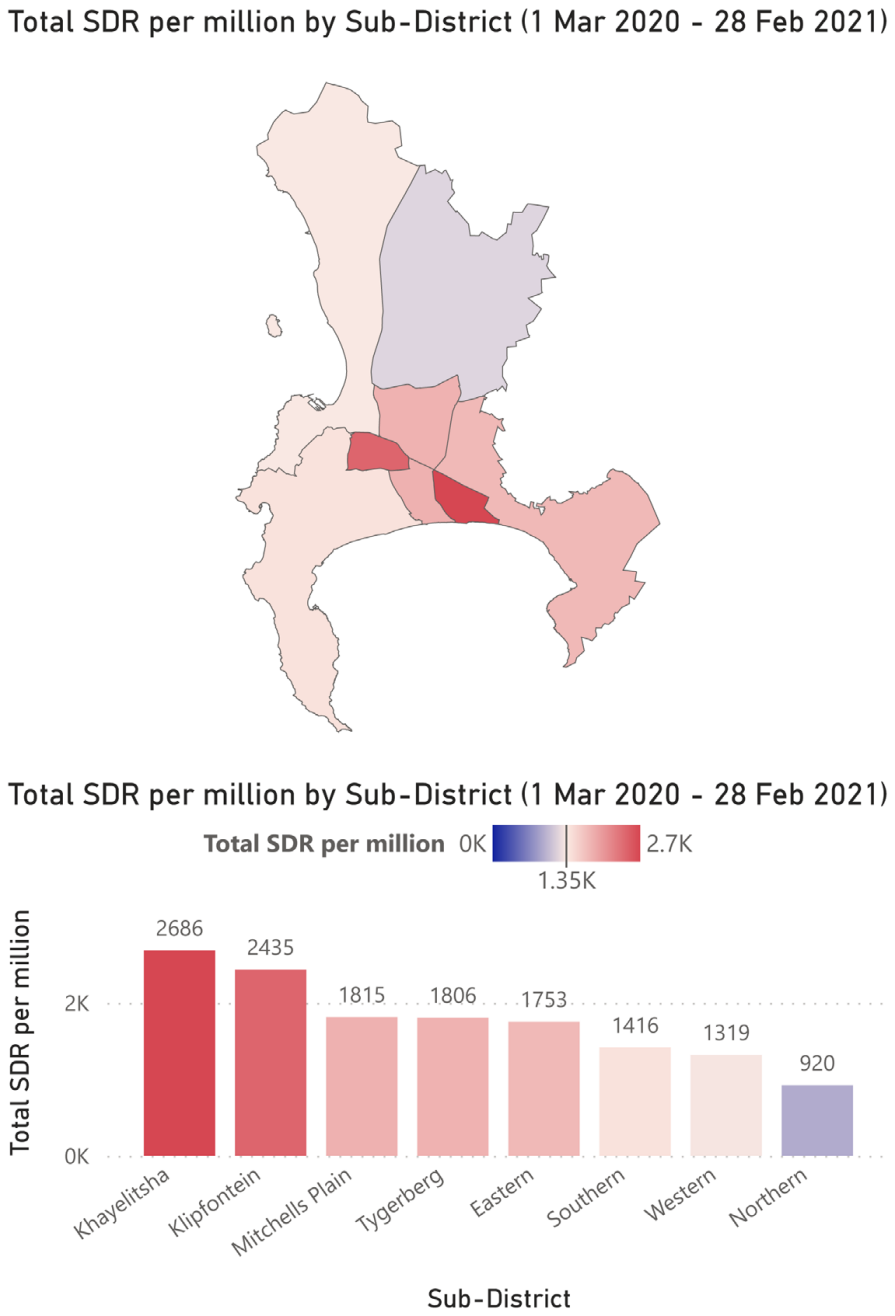
Total COVID-19 Standardized Death Rate (SDR) per million by sub-district. Graphic courtesy of the Western Cape Department of Health.

There were 7 643 total COVID-19 related deaths in the Cape Town Metro. The total crude death rate ranged from 1 217 in Northern, to 2 547 in Klipfontein. Adjusted for age, the total standardized death rate for COVID-19 was 1 640 per million overall but ranged from 920 in Northern to 2 686 in Khayelitsha sub-district ( Table 1, Figure 2).

**Table 1.** Total Number of COVID-19 deaths, Crude Death Rate (CDR) per million and Standardized Death Rate (SDR) per million in the Cape Town Metro, in each wave and by sub-district.

	Wave 1			Wave 2			Total			
	1 Mar - 31 Oct 2020			1 Nov - 28 Feb 2021			1 Mar - 28 Feb 2021			Ratio of Wave 2 to Wave 1 SDR
Sub-district	Total deaths	CDR per million	SDR per million	Total deaths	CDR per million	SDR per million	Total deaths	CDR per million	SDR per million	
**Eastern**	454	645	656	754	1070	1097	1208	1715	**1753**	1.67
**Khayelitsha**	404	913	1605	257	581	1080	661	1493	**2686**	0.67
**Klipfontein**	530	1292	1234	515	1255	1201	1045	2547	**2435**	0.97
**Mitchells** **Plain**	376	611	793	496	806	1022	872	1417	**1815**	1.29
**Northern**	195	417	313	374	800	608	569	1217	**920**	1.94
**Southern**	437	732	549	685	1148	867	1122	1880	**1416**	1.58
**Tygerberg**	503	689	695	797	1092	1110	1300	1781	**1806**	1.60
**Western**	349	553	529	517	819	790	866	1372	**1319**	1.49
**METRO Total**	**3248**	**706**	**696**	**4395**	**956**	**943**	**7643**	**1662**	**1640**	**1.35**

**Figure 3a and 3b.**
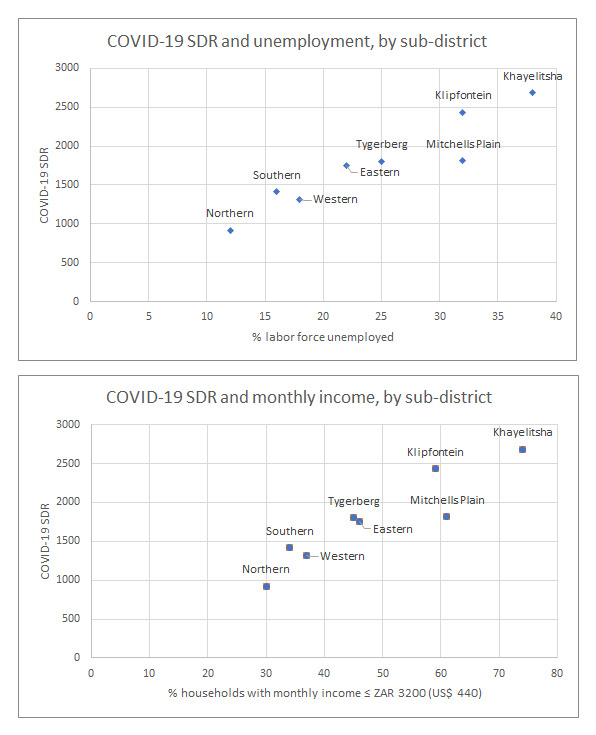
Total COVID-19 Standardized Death Rate (SDR) and percent of labor force unemployed ( **a**) and household monthly income ( **b**), taken from the 2011 Census ^3^, and plotted for each Cape Town Metro sub-district.

Using the 2011 Census data, the eight sub-districts have differing levels of employment and monthly household income. Among the deceased COVID-19 cases, more private laboratory testing was done in the higher income sub-districts ( Table 2). The scatter plots ( Figure 3a and b) show a linear positive relationship between increasing COVID-19 SDR in a sub-district and (a) percentage of unemployment and (b) percentage of low-income households in a sub-district.

**Table 2.** Economic indicators (taken from 2011 Census ^3^) and test facility for deceased COVID-19 cases, by sub-district.

Sub-district	% Labor force (aged 15-64) unemployed	% Households with monthly income ≤ ZAR3200	% Private testing for deceased COVID-19 cases
Eastern	22	46	36.3
Khayelitsha	38	74	14
Klipfontein	32	59	23.9
Mitchells Plain	32	61	20.5
Northern	12	30	54.9
Southern	16	34	42.6
Tygerberg	25	45	29.7
Western	18	37	41.2

**Figure 4.**
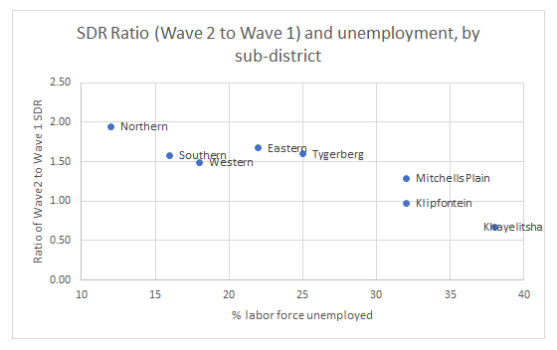
Ratio of Wave 2 to Wave 1 COVID-19 SDRs and percent of labor forced unemployed, by sub-district.

Poorer sub-districts tended to be worse affected in the first wave with relative protection in the second wave. In contrast, wealthier sub-districts had a low SDR during the first wave but were worse affected in the second wave. A linear but inverse relationship was present between percentage unemployment rate in a sub-district and the ratio of Wave 2 to Wave 1 COVID-19 SDRs ( Figure 4).

**Figure 5.**
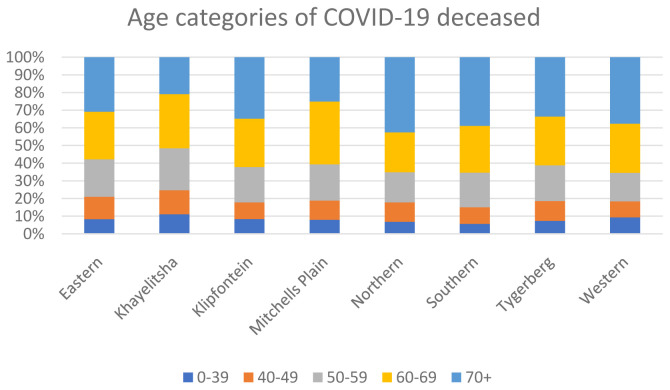
Age categories for the deceased COVID-19 patients, by sub-district.

**Figure 6.**
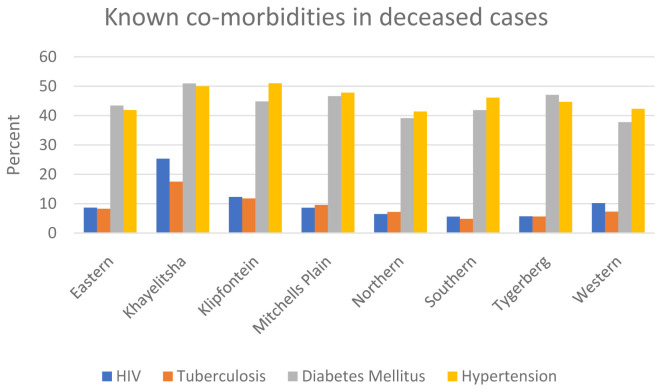
Known co-morbidities in deceased COVID-19 patients, by sub-district (note that tuberculosis includes both previous and current disease).

For the deceased patients with COVID-19, different sub-districts also have differing age structures and co-morbidity burdens. Sub-districts with higher burdens of infectious diseases, particularly HIV and tuberculosis, tended to have younger COVID-19 deaths and an overall higher SDR ( Figure 5 and Figure 6).

## Discussion

The study suggests that low-income sub-districts had higher COVID-19 SDRs. The SDR for Khayelitsha was almost three times higher than that of Northern, a more affluent sub-district. This finding of a strong socio-economic gradient in COVID-19 mortality is consistent with an expanding literature, and explanations for this association are multifactorial. Individuals in low-income communities with crowded housing, a reliance on public transport and higher numbers of essential workers are less able to implement and maintain social distancing and non-pharmaceutical interventions (NPIs) ^7^. And they therefore are more likely to get infected with COVID-19, as demonstrated in several South African seroprevalence studies ^15– 17^.

A SARS-CoV-2 seroprevalence study performed by the South African blood service in four of the nine provinces (Eastern Cape, Northern Cape, Free State and KwaZulu Natal) found substantial differences between different race groups, with Black African donors having seroprevalence rates above 60% and White donors having less than or around 20% ^15^. While the authors note the limitations of using race as a proxy for socio-economic status, their study highlights the limitations of NPIs in communities with economic deprivation and high population density, or conversely how effective NPIs can be when they are feasible ^15^. A study on shopping mall workers in Cape Town found a high seroprevalence was associated with informal housing, living in a sub-district with a low-income per household and having a low-earning occupation ^16^.

In a study from the USA, amongst those diagnosed with COVID-19, poverty was also associated with a higher risk of hospitalization and intensive care unit admission ^18^. Because of upstream social determinants of health, low-income communities have higher rates of chronic diseases that put them at risk of severe COVID-19 disease ^7^. Younger age is usually a protective factor for severe COVID-19 disease. However, data from the USA and UK has shown that in communities of color, people of younger ages were still at risk of COVID-19 mortality, because high rates of co-morbidities, such as diabetes and chronic lung , are concentrated in these poor communities ^19^. The diagnosis and management of these chronic conditions is also inferior, as these same communities often lack access to quality care ^20^.

Data from a population-based study in the Western Cape during the first wave of the pandemic showed that older age, male sex and diabetes were strongly associated with COVID-19 mortality ^12^. The study also found that HIV and current tuberculosis, both conditions strongly associated with poverty, were associated with COVID-19 mortality, with a 2.14 (95% confidence interval [CI] 1.70-2.70) and 2.70 (95% CI 1.81-4.04) adjusted hazard ratio, respectively ^12^. A population-based study from the UK also found a strong association between HIV and COVID-19 mortality ^21^.

As data on co-morbidities is not collected in a standardized way across the public and private sectors for all cases diagnosed with COVID-19, this study was not able to formally assess the differing burdens of co-morbidities across the sub-districts.

If individuals in low-income communities require in-patient treatment, they are more likely to access the relatively under-resourced public health sector. An analysis of COVID-19 in-hospital mortality in South Africa, found that admission to a public sector compared to a private sector facility was associated with increased risk of mortality (adjusted odds ratio of 1.6; 95% CI 1.4-1.8) ^22^. This may be due to later presentations to hospital, as well as differences in access to and unequal availability of critical care and other specialized resources or interventions in the public sector.

This study also showed that low-income sub-districts were worse affected in the first wave compared to the second wave, suggesting that infection in the first wave may have conferred some immunity. However, the science on re-infection is still very unclear, and particularly in the context of emerging variants, this should be interpreted cautiously ^23^.

Limitations of this study include its ecological nature, the lack of standardized co-morbidity data and the fact that the economic data is from 2011. However, while the percentages of individuals living in poverty in each sub-district might have changed with time, and even increased during the pandemic, a reduction in inequality between the sub-districts is unlikely to have occurred. The Western Cape Government reported increasing inequity in Cape Town from 2011 to 2018, with the Gini coefficient for the city increasing from 0.604 in 2011 to 0.617 in 2018 ^11^. An analysis of data from the National Income Dynamics Study (NIDS) in 2017 and the first wave of the NIDS-Coronavirus Rapid Mobile Survey (NIDS-CRAM) suggested that income-related health inequality in the COVID-19 era increased six-fold compared with what was obtained in 2017 ^24^.

The COVID-19 pandemic has exposed the longstanding structural drivers of health inequities globally ^25^. While all sectors of the population of Cape Town were affected by the pandemic, those living in low-income communities were at higher risk of SARS-CoV-2 infection and of COVID-19-related mortality. Health services need to be preferentially directed to those that need them the most, i.e. the poor. This is not only for COVID-19, but for any subsequent pandemic where low-income communities will remain vulnerable without improved access to care. It will however remain difficult to address the inequities of COVID-19 morbidity and mortality while poverty and income inequality persist.

## Data availability

All data underlying the results are available as part of the article and no additional source data are required.

## References

[1] World Bank: Gini index (World Bank estimate).2021 [cited 2021 Apr 12]. Reference Source

[2] International Bank for Reconstruction and Development / The World Bank: Overcoming Poverty and inequality in South Africa - An assessment of Drivers, Constraints and Opportunities.2018. Reference Source

[3] Statistics South Africa: Cape Town census and population statistics.2011. Reference Source

[4] Western Cape Government: Socio-economic profile. City of Cape Town.2017. Reference Source

[5] Tegally H Wilkinson E Giovanetti M: Emergence and rapid spread of a new severe acute respiratory syndrome-related coronavirus 2 (SARS-CoV-2) lineage with multiple spike mutations in South Africa. *medRxiv.* 2020;2020.12.21.20248640. 10.1101/2020.12.21.20248640

[6] Jassat W Cohen C Mudara C: Multivariable analysis comparing in-hospital mortality in the first and second wave of Covid-19 in three districts of South Africa. *Covid-19 Spec Public Heal Surveill Bull.* 2021;18(6). Reference Source

[7] Karmakar M Lantz PM Tipirneni R: Association of Social and Demographic Factors With COVID-19 Incidence and Death Rates in the US. *JAMA Netw Open.* 2021;4(1):e2036462. 10.1001/jamanetworkopen.2020.36462 33512520 PMC7846939

[8] Office for National Statistics: Coronavirus (COVID-19) related deaths by ethnic group, England and Wales - : 2 March 2020 to 10 April 2020.2020 [cited 2021 Mar 4]. Reference Source

[9] Millán-Guerrero RO Caballero-Hoyos R Monárrez-Espino J: Poverty and survival from COVID-19 in Mexico. *J Public Health (Oxf).* 2020;fdaa228. 10.1093/pubmed/fdaa228 33367803 PMC7798985

[10] Martins-Filho PR de Souza Araújo AA Quintans-Júnior LJ: COVID-19 fatality rates related to social inequality in Northeast Brazil: a neighbourhood-level analysis. *J Travel Med.* 2020 [cited 2021 Feb 25];27(7):taaa128. 10.1093/jtm/taaa128 32761125 PMC7454826

[11] Western Cape Government - Health: Western Cape Burden of Disease - Rapid Review Update 2019.2020. Reference Source

[12] Boulle A Davies MA Hussey H: Risk factors for COVID-19 death in a population cohort study from the Western Cape Province, South Africa. *Clin Infect Dis.* 2020 [cited 2021 Feb 3];ciaa1198. 10.1093/cid/ciaa1198 32860699 PMC7499501

[13] South African Reserve Bank: Selected Historical Rates.[cited 2021 Apr 14]. Reference Source

[14] Boulle A Heekes A Tiffin N: Data centre profile: The provincial health data centre of the western cape province, South Africa. *Int J Popul Data Sci.* 2019;4(2):1143. 10.23889/ijpds.v4i2.1143 32935043 PMC7482518

[15] Sykes W Mhlanga L Swanevelder R: Prevalence of anti-SARS-CoV-2 antibodies among blood donors in Northern Cape, KwaZulu-Natal, Eastern Cape, and Free State provinces of South Africa in January 2021. *Res Sq.* 2021 [cited 2021 Feb 25];rs.3.rs-233375. 10.21203/rs.3.rs-233375/v1 33594353 PMC7885925

[16] Shaw JA Meiring M Cummins T: Higher SARS-CoV-2 seroprevalence in workers with lower socioeconomic status in Cape Town, South Africa. Musuka G, editor. *PLoS One.* 2021 [cited 2021 Mar 1];16(2):e0247852. 10.1371/journal.pone.0247852 33630977 PMC7906413

[17] Hsiao M Davies MA Kalk E: SARS-CoV-2 seroprevalence in the Cape Town metropolitan sub-districts after the peak of infections. *Covid-19 Spec Public Heal Surveill Bull.* 2020;18(Supplementary Issue 5). Reference Source

[18] Muñoz-Price LS Nattinger AB Rivera F: Racial Disparities in Incidence and Outcomes Among Patients With COVID-19. *JAMA Netw Open.* 2020;3(9):e2021892. 10.1001/jamanetworkopen.2020.21892 32975575 PMC7519420

[19] Klugman KP Zewdu S Mahon BE: Younger ages at risk of Covid-19 mortality in communities of color [version 1; peer review: 2 approved]. *Gates Open Res.*F1000 Research Ltd;2020;4:69. 10.12688/gatesopenres.13151.1 32715283 PMC7360879

[20] Kruk ME Gage AD Arsenault C: High-quality health systems in the Sustainable Development Goals era: time for a revolution. *Lancet Glob Health.*Elsevier Ltd;2018;6(11):e1196–e1252. 10.1016/S2214-109X(18)30386-3 30196093 PMC7734391

[21] Bhaskaran K Rentsch CT MacKenna B: HIV infection and COVID-19 death: a population-based cohort analysis of UK primary care data and linked national death registrations within the OpenSAFELY platform. *Lancet HIV.* 2021;8(1):e24–e32. 10.1016/S2352-3018(20)30305-2 33316211 PMC7773630

[22] Jassat W Mudara C Ozougwu L: Increased mortality among individuals hospitalised with COVID-19 during the second wave in South Africa. *medRxiv.* 2021;2021.03.09.21253184. 10.1101/2021.03.09.21253184

[23] Cele S Gazy I Jackson L: Escape of SARS-CoV-2 501Y.V2 variants from neutralization by convalescent plasma. *medRxiv.* 2021;2021.01.26.21250224. 10.1101/2021.01.26.21250224 PMC9867906 33780970

[24] Nwosu CO Oyenubi A: Income-related health inequalities associated with the coronavirus pandemic in South Africa: A decomposition analysis. *Int J Equity Health.* 2021 [cited 2021 Apr 21];20(1):21. 10.1186/s12939-020-01361-7 33413442 PMC7790046

[25] Paremoer L Nandi S Serag H: Covid-19 pandemic and the social determinants of health. *BMJ.*BMJ Publishing Group;2021;372:n129 . 10.1136/bmj.n129 33509801 PMC7842257

