# Supplementary material for: Higher COVID-19 mortality in low-income communities in the City of Cape Town – a descriptive ecological study
Source: Gates Open Res. 2021 Jun 4;5:90. [Version 1] doi: 10.12688/gatesopenres.13288.1 (PMC8264385; doi:10.12688/gatesopenres.13288.1)
Supplement: Peer review history

# Peer review: Reviewer response for version 1

**DOI:** 10.21956/gatesopenres.14527.r30740

**Published:** 2021-07-07

**Authors:** Jeanelle de Gruchy

**Affiliations:** 1 Tameside Council, Ashton-under-Lyne, UK; 2 Association of Directors of Public Health (UK), Ashton-under-Lyme, UK

A descriptive ecological study with routine data showing the association of COVID-19 mortality and economic inequality in Cape Town. An important, timely study. Methods, strengths and limitations are well set out and discussed. 

 The difference between SDR in the first and second waves is difficult to explain, especially as it is not in line with the main finding. Would there have been a sufficiently high enough population infected in the first wave to provide protection to that extent in the second wave? (the seroprevalence study gives some indication that it is high in these communities, but it's unclear what period the study covered, and its limitation is it wasn't in the Western Cape).  It would be good to explore alternate explanations to the one already offered.

 In the discussion of in-patient treatment, it would be helpful to provide information of the proportionate use of public and private sector in the sub-districts. 

 The final paragraph rightly focuses on equitable access to health services. It would be good to consider whether there are any other recommendations to mitigate the high exposure and transmission in these communities, e.g. how effective were non-pharmaceutical interventions, e.g. did essential workers have access to personal protective equipment. For subsequent health threats, tackling the high levels of obesity and non-pharmaceutical diseases would also be important. Finally, inequitable access to vaccination is likely to be a critical driver of higher COVID-19 SDRs in poorer communities.   

 Minor points:

- Introduction section, 3rd paragraph footnote for findings in the UK should only be reference 8? 
- Methods section, 3rd paragraph last sentence meaning is unclear. 

Is the work clearly and accurately presented and does it cite the current literature?

Yes

If applicable, is the statistical analysis and its interpretation appropriate?

I cannot comment. A qualified statistician is required.

Are all the source data underlying the results available to ensure full reproducibility?

Yes

Is the study design appropriate and is the work technically sound?

Yes

Are the conclusions drawn adequately supported by the results?

Yes

Are sufficient details of methods and analysis provided to allow replication by others?

Yes

Reviewer Expertise:

Public health expert with extensive experience of managing COVID-19 in the UK.

I confirm that I have read this submission and believe that I have an appropriate level of expertise to confirm that it is of an acceptable scientific standard.

# Peer review: Reviewer response for version 1

**DOI:** 10.21956/gatesopenres.14527.r30741

**Published:** 2021-06-10

**Authors:** Keith P. Klugman

**Affiliations:** 1 Bill & Melinda Gates Foundation, Seattle, WA, USA

This article provides an ecological analysis of poverty and lack of access as associated with increased COVID mortality in Cape Town, South Africa.

 The strengths and limitations of the study design are well discussed. 

 The lack of access to COVID diagnostics in the private sector by poorer communities may have substantially reduced the apparent numbers of COVID deaths and reduced the differences recorded in this study - the study therefore is likely a minimal assessment of the magnitude of poverty on COVID mortality risk - this point could be added to the discussion. 

 The significantly higher attack rate in Khayalitsha in the first wave relative to the second does suggest some degree of longer term protection - given the importance of the beta COV in the second wave this deserves some additional comment - there are some data to suggest that although short term exposure to wave 1 infection may not be protective from wave 2, that longer term antibodies which are affinity matured may be more protective, as suggested in this analysis.

 Given the bias against testing in poorer communities, have the authors been able to look at the relationship between wealth of districts and all cause excess mortality during these time periods of maximal virus circulation?

Is the work clearly and accurately presented and does it cite the current literature?

Yes

If applicable, is the statistical analysis and its interpretation appropriate?

Yes

Are all the source data underlying the results available to ensure full reproducibility?

Yes

Is the study design appropriate and is the work technically sound?

Yes

Are the conclusions drawn adequately supported by the results?

Yes

Are sufficient details of methods and analysis provided to allow replication by others?

Yes

Reviewer Expertise:

Infectious diseases, epidemiology and microbiology

I confirm that I have read this submission and believe that I have an appropriate level of expertise to confirm that it is of an acceptable scientific standard.
